# Testing preference of alfalfa hay of different relative feed value and brome hay in lactating Jersey cows

**DOI:** 10.3168/jdsc.2024-0653

**Published:** 2024-10-29

**Authors:** K.K. Buse, P.J. Kononoff

**Affiliations:** Department of Animal Science, University of Nebraska–Lincoln, Lincoln, NE 68503

## Abstract

Forages comprise a large portion of dairy cattle rations, and quality can have a large influence on feed intake and milk production. Although factors like particle size, fiber content, and digestibility affect it, palatability can also play a role in feed intake. The objective of this study was to conduct an experiment to evaluate the effect of relative feed value (RFV) on feed preference. A taste preference sequential elimination experiment using 16 lactating Jersey cows (210 ± 4 DIM, 27.4 ± 3.24 kg/d milk yield, 19.6 ± 1.52 kg/d DMI; ± SD) was conducted to test this objective. Reduced-lignin alfalfa hay diets with a high RFV (213; HIRFV), mid RFV (163; MDRFV), and low RFV (94; LORFV) were compared with a control smooth brome grass hay (75 RFV; Brome). Cows were offered 0.45 kg of each alfalfa treatment and 0.23 kg of the hay treatment in a randomized arrangement within the feed bunk for 1 h or until the feed was fully consumed or consumed in the greatest amount. Feeds were offered for 9 d with all treatments offered the first 4 d. The most preferred feed was then removed, and the remaining 3 feeds were offered for 3 d. The process was repeated for the last 2 d. Feed preference was ranked from 1 to 4 with 1 being the most preferred and 4 the least; this was in the order they were removed. The rankings of each treatment for each cow were summed and averaged. The probability of a treatment being chosen first was determined using the Plackett-Luce model. The preference ranking of HIRFV was highest (1.06 ± 0.25), followed by LORFV (2.56 ± 0.63), MDRFV (2.75 ± 0.58), and Brome (3.25 ± 0.93). The probability of HIRFV being selected first is 95.7% ± 0.79%, whereas the probabilities of LORFV, MDRFV, and Brome being selected first are 2.09% ± 0.38%, 1.70% ± 0.38%, and 0.55% ± 0.46%, respectively. These results support the notion that higher quality alfalfa hay is preferred by cows. The secondary preference of LORFV could be associated with the presence of byproducts of Maillard reactions, which would lower the RFV while providing more favorable flavors than were present in MDRFV and Brome.

## Highlights

• We examined feed preference of 3 alfalfa hays differing in RFV and 1 brome hay.
• Cows clearly most preferred the highest-quality alfalfa.
• Cows exhibited a slight preference for lower RFV alfalfa over mid-RFV alfalfa.
• The preference for low over mid RFV may have been in part due to Maillard reaction compounds.
• Smooth brome grass hay was least preferred.

## Graphical Abstract

**Figure:**
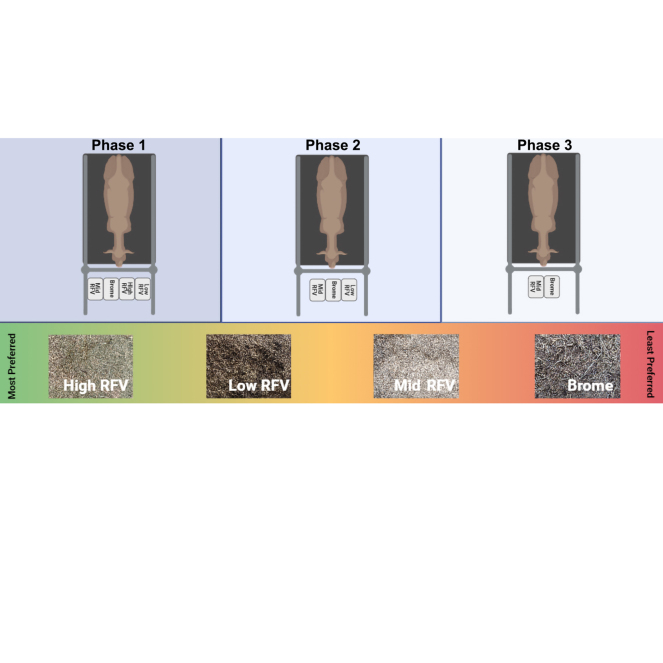
**Summary:** This article describes an experiment conducted to determine the preference of forage differing in relative feed value (RFV). Cows were offered 3 different alfalfa hays with RFV of 213, 163, and 94, as well as a brome grass hay with an RFV of 75. Alfalfa hay with a high RFV was most preferred, followed by low-RFV alfalfa hay, and brome grass hay was the least preferred. We speculate that the low-RFV alfalfa hay outranked mid-range RFV alfalfa hay due to product formation during Maillard reactions. Created with BioRender.com.

Dry matter intake is the key factor that influences milk production in dairy cattle, and feed characteristics such as preference can influence feed intake (Grant and Albright, 2000). Forages play a crucial role in dairy cattle rations, often comprising 50% or more of a typical ration (Kendall et al., 2009), and thus many physical and chemical properties of these feeds stand to have a major influence on overall feed preference. In the United States, corn silage is the most prominent forage of these diets, but alfalfa hay is also often included. The quality of alfalfa hay can be affected by factors ranging from maturity at harvest to moisture content at baling. Forage quality can be defined as the potential of a forage to elicit a desired production response. Consequently, because it affects DMI and in turn milk yield, an animal's willingness to consume feed can be one measure of forage quality. Plant-based factors may affect the cow's preference for a particular feed, which may include leafiness, moisture content, and natural plant compounds (Ball et al., 2001). Forage quality can described using different calculations and indexes, but commercially, relative feed value (**RFV**) is the index commonly used to compare the quality of forages and is largely based on the NDF and ADF content (Jeranyama and Garcia, 2004).

Although there is no universally recognized definition of palatability (Marten, 1978), palatability of a forage is determined by appeal of forages to stimulate the sense of taste, odor, and texture in animals (Gherardi and Black, 1991). Cattle process sensory information, primarily taste and smell but also sight, allegedly to determine if harmful compounds are present in feedstuffs (Provenza, 1995; Grant and Albright, 2000). Even though the feeds commonly fed to dairy cattle rarely pose harm, cattle still use discerning behaviors to select feeds to consume. Studies examining the dairy cattle feed preference of forages have mostly been completed in calves and heifers (Marten, 1978; Undi and Wittenberg, 1996; Erickson et al., 2012; Kargar and Kanani, 2019) or grazing cattle (Ridley et al., 1963; Lombardi et al., 2015). Leonardi and Armentano (2003) did test the effect of quality, among other characteristics, of alfalfa hay on the selective consumption of lactating dairy cattle, and they observed no difference in consumption between lower quality and higher quality alfalfa hay. In their study, alfalfa hay was offered in a TMR instead of in isolation, and as a result, interacting feed factors could have influenced preference. To our knowledge, no preference studies have been conducted evaluating alfalfa hay alone. For this reason, the objective of this study was to evaluate cattle preference for alfalfa hays varying in RFV. We hypothesized that cattle would prefer hay with a higher RFV most and those with a lower RFV less.

Animal care and experimental procedures were approved by the University of Nebraska–Lincoln Animal Care and Use Committee. Sixteen lactating Jersey cows (210 ± 4 DIM, 27.4 ± 3.24 kg/d milk yield, 19.6 ± 1.52 kg/d DMI; ± SD) were used to complete the taste preference experiment in a manner described by Erickson et al. (2004) and Carroll et al. (2023). The cows were housed in individual tiestalls equipped with rubber mats in a temperature-controlled (20°C) barn with continuous access to water at the Dairy Metabolism Facility in the Animal Science Complex at the University of Nebraska–Lincoln. Cows were milked at 0700 and 1800 h each day. Animals were fed a TMR once daily. On a DM basis this diet contained 52.6% forage (38.5% corn silage, 14.1% alfalfa hay) and 47.4% grain and supplement mix (15.9% ground corn, 10.3% corn distillers grains and solubles, 9.40% soybean meal, 2.82% expellers soybean meal, 1.79% soyhulls, 1.88% fat supplement, 1.24% molasses, 1.39% calcium carbonate, 0.64% sodium bicarbonate, 0.56% dicalcium phosphate, 0.49% salt, 0.26% magnesium oxide, 0.41% rumen-protected lysine, 0.26% rumen-protected methionine, and 0.08% vitamin and trace mineral supplement). On a DM basis this diet contained 17.3% CP, 26.04% NDF, 25.6% starch, and 4.9% crude fat. Before feeding the daily allotment of a TMR, cows were offered 0.45 kg of each treatment in a randomized arrangement and separated within 30.5 cm × 40.6 cm plastic tubs. Treatments were offered from 1000 to 1100 h or until a single treatment was fully consumed, whichever came first. Treatments were offered for 9 d total as described with all 4 treatments allocated from d 1 to 4. After the initial feeding period, the most preferred feed for each individual cow was removed, and 3 feeds were offered for 3 d. The process was repeated for 2 d.

Treatments were ranked sequentially with 1 being the most preferred and 4 being the least. This is the same way in which they were removed during the experiment. The 4 hays examined included a high-RFV alfalfa hay (**HIRFV**; 213 RFV), a mid-range RFV alfalfa hay (**MDRFV**; 163 RFV), a low-RFV alfalfa hay (**LORFV**; 94 RFV), and a brome grass hay (**Brome**; 75 RFV). All alfalfa hays were harvested during the 2020 growing season from the same field, and the conditions under which those hays were grown are described in Buse et al. (2024). The LORFV and MDRFV hay bales were from the second cutting, which was cut on July 6, and the HIRFV hay bale came from the fourth cutting, which was cut on September 18. The hay was baled into large round bales and stored for 9 to 11 mo in a covered, 3-sided commodity shed with adequate airflow to prevent overheating. The brome hay was grown the same year on a nearby field. A subsample of the core samples from each bale were ground to pass a 1-mm screen (Wiley Mill, Arthur A Thomas Co., Philadelphia, PA) and analyzed by near infrared spectroscopy by Cumberland Valley Analytical Services Inc. (Waynesboro, PA) for DM, CP, acid detergent insoluble N, neutral detergent insoluble N, ADF, NDF, lignin, starch, crude fat, and ash. Relative feed value was calculated using the equation (Jeranyama and Garcia, 2004)

RFV = {[88.9 – (0.799 × % ADF)] × (120/% NDF)}/1.29.

A single bale was chosen for each treatment, and this was based on their representation of the 2 extremes and average of the sample population. Selected bales were ground to pass through a 200-mm screen (Mighty Giant model 2015 PTO, Jones Manufacturing, Beemer, NE). Each bale was ground into a pile separate from the other bales with adequate grinding time during the transition to the next bale to prevent any cross contamination. Ground hay was stored in piles for 7 d before a portion of each pile was collected into individual trash cans with a secured lid (Rubbermaid, Wooster, OH) and transported to the Animal Science Complex at the University of Nebraska–Lincoln. Each hay type was manually select daily before being weighed into the plastic tubs to ensure a representative sample was used. To collect a representative portion and to minimize loss of fine particles, a feed shovel was used to manually transfer mixed hay to the tubs.

The mean and SD of treatment rankings were calculated using the PROC CORR function of SAS (version 9.4; SAS Institute Inc.). Data were also analyzed with the Plackett-Luce model in R (version 1.3.1093; https://www.r-project.org/) to estimate the probability an examined treatment would be chosen first based on the rankings from the current dataset. A Z test was conducted in R on the probabilities to determine if the probability differed from the probability of no preference at 25% (100/4; Fligner and Verducci, 1988). Significance was declared with a *P*-value ≤0.05.

Daily feed intake averaged 20.5 ± 2.83 kg (mean ± SD) and milk yield averaged 27.0 ± 2.81 kg (mean ± SD; data not shown). Table 1 summarizes the chemical composition of the treatment alfalfa hays and brome grass hay. Relative feed value is influenced by the ADF and NDF content, and as both values increase, RFV decreases (Jeranyama and Garcia, 2004). This response is observed as the ADF increased from 24.9% to 44.1% of DM and NDF increased from 30.4% to 67.8% of DM across treatments to result in RFV ranging from 213 for HIRFV to 75 for Brome. The CP content of the treatment hays increased from 10.0% for Brome to 22.3% for HIRFV as RFV increased.

**Table 1:** Chemical composition of alfalfa hay and brome grass hay (% of DM)^1^

Item	LORFV	MDRFV	HIRFV	Brome
DM, % as is	89.2	91.6	92.2	91.7
CP	19.3	21.7	22.3	10.0
Soluble protein	1.30	6.70	8.10	2.40
ADIN	3.15	1.54	1.42	1.51
NDIN	10.2	3.88	2.91	4.06
NDF	54.6	37.1	30.4	67.8
aNDFom	49.2	35.6	29.3	61.9
ADF	43.0	30.7	24.9	44.1
Lignin	9.77	6.94	5.51	6.09
NFC	17.3	30.4	36.3	9.41
Ash	15.7	11.7	11.1	9.41
RFV	94	163	213	75

1 n = 2. LORFV = low relative feed value alfalfa hay; MDRFV = mid-range relative feed value alfalfa hay; HIRFV = high relative feed value alfalfa hay; Brome = brome grass hay; RFV = relative feed value; aNDFom = NDF conducted with α-amylase and corrected for organic matter.

Preference results are listed in Table 2. The treatment with the best ranking, thus being the most preferred, was HIRFV (1.06; SD = 0.25) followed by LORFV (2.56; SD = 0.63), MDRFV (2.75; SD = 0.58), and Brome (3.25; SD = 0.93). Fifteen of the 16 cows indicated HIRFV as their most preferred choice during the first feeding segment with the exception of one cow selecting Brome. The results from the Plackett-Luce model and corresponding Z test are summarized in Table 2. This measure expresses the probability that a treatment will be chosen first if all the treatments were available. The probability was the highest for HIRFV with 95.7% ± 0.79%, then LORFV at 2.09% ± 0.38% followed by MDRFV at 1.70% ± 0.38% and Brome at 0.55% ± 0.46%. High RFV alfalfa hay had a Z test score of 4.13, meaning that the probability HIRFV is selected is greater than the chance of selection if there was no preference or a 25% chance of being chosen. The Z test scores for LORFV, MDFRV, and Brome were −1.48, −2.03, and −4.16, respectively, meaning the chance of the remaining treatments being selected is less than 25%.

**Table 2:** Preference scores of 4 forages different by species and relative forage value (RFV) in lactating Jersey cattle and the probability animals will choose a given treatment

Item	Treatment^1^			
	LORFV	MDRFV	HIRFV	Brome
Mean rank (SD)	2.56 (0.63)	2.75 (0.58)	1.06 (0.25)	3.25 (0.93)
μ^2^	2.09	1.70	95.7	0.55
SE^3^	0.38	0.38	0.79	0.46
Z^4^	−1.48	−2.03	4.13	4.16
*P*-value	0.138	0.043	<0.001	<0.001

1 HIRFV = high RFV alfalfa hay; MDRFV = mid-range RFV alfalfa hay; LORFV = low RFV alfalfa hay; Brome = brome grass alfalfa hay.

2 μ = the estimated percent chance that a treatment will be chosen first when all 4 treatments are offered together.

3 SE = standard error for the chance a treatment will be chosen first.

4 Z tests whether μ is different from 25% (the percent chance a treatment will be chosen if there were no preferences).

Cattle make preference decisions based upon on the senses of sight, touch, smell, and taste, and they are believed to have excellent memory associated with each of these senses (Marino and Allen, 2017). Previous research (Mayland et al., 2005) has compared the effect of cutting time and associated the presence of some plant compounds with feed preference. These plant compounds indicated desirable forage components associated with hays that the animals had previously been exposed to and selected. It is possible that cattle selected hay based upon circumstance and feed characteristics from past experience, which could explain why HIRFV was the most preferred treatment (Mayland et al., 2005). Because hay can vary in quality, cattle are often exposed to a wide variety of hay and are likely able to discern hay quality based on past experiences. In the instance of the Mayland et al. (2005) study, cattle preferred afternoon-cut hay due to its higher sugar content. Cattle have approximately 20,000 taste buds and can distinguish the 4 primary tastes of sweet, salty, bitter, and sour. They are believed to have a high preference for sweet flavors likely because this characteristic is associated with a high caloric value (Marino and Allen, 2017). Conversely, cattle avoid bitter-tasting feeds because they could potentially contain toxic compounds (Marten, 1978; Marino and Allen, 2017). High RFV hay contains a higher concentration of NFC due to less sugars lost through prolonged respiration and Maillard reactions (Rotz and Muck, 1994). In the current study, we observed that most of the cattle smelled each feed offering before making a choice during the first 2 to 3 d of the study. We speculate that cows used their sense of smell to discern the presence of high levels of NFC in the HIRFV sample, which they then associated with most preferable.

Unexpectedly, we observed that cattle preferred LORFV slightly more than MDRFV alfalfa hay, making it the second most preferred treatment. When bales contain a high moisture content, spontaneous heating can occur. The heating can result in subsequent products formed through Maillard reactions that can appear as lignin-like during sequential detergent analysis; the resulting products can be indicated by the forage's ADIN (Rotz and Muck, 1994; Ball et al., 2001; Collins et al., 2018). During Maillard reactions, carbohydrates, mostly sugars, are polymerized with amino acids to yield products that are not degraded in the rumen (Sindt et al., 2004) and, at least to the human experience, possess a nutty, sweet flavor (Starowicz and Zieliński, 2019). As previously discussed, cattle are drawn to sweet-tasting feeds. A higher concentration of ADIN in LORFV compared with the other treatments indicates a higher concentration of Maillard reaction products, and we speculate this influenced feed preference.

It is important to note that brome and alfalfa are different forage species with their own distinct nutritional profiles and characteristics. Thus, comparison of the RFV values alone can be misleading; more estimates of chemical composition are needed for accurate comparison. Between previous exposure to grass hay and association with lower nutritional value as well as a less enticing taste and smell, other treatments appeared to be preferred over Brome. Additionally, cattle are very sensitive to touch and have mechanical touch and thermal receptors in the muzzle as well as nociceptors, which are the cells that initiate the pain signal (McCleskey and Gold, 1999), that respond to damaging and potentially damaging stimuli (Marten, 1978; Marino and Allen, 2017). Cattle use their sense of touch to search out the more desired parts of plants, preferring leaves over stems (Arnold, 1966). Although we do not understand precisely why cattle least preferred Brome, the leaf-to-stem ratio difference between alfalfa and grass hay could also help explain why Brome was the least preferred treatment.

In summary, a taste preference experiment evaluating cattle preference for alfalfa hays of diffing RFV was conducted. Results indicate that cows prefer high-quality alfalfa hay over lower quality alfalfa hay. This is likely due to the initial impact the sense of smell has on the decision of what cows consume first. Even though the probability of selection is similar between mid-range and low-quality alfalfa hay, low-quality alfalfa hay appears to be more preferred, and we speculate that may be due to products formed from Maillard reactions.
